# Skinny (lower) legs run fast marathons

**DOI:** 10.1113/EP093503

**Published:** 2025-12-08

**Authors:** Alexander C. Berry, L. Bruce Gladden

**Affiliations:** ^1^ School of Kinesiology Auburn University Auburn Alabama USA

**Keywords:** endurance performance, oxygen uptake, running economy

1

Male Kenyan runners currently have 12 of the fastest 5 times in foot races from 800 m to the marathon, and 21 of the fastest 10 times in the same races (www.worldathletics.org). This represents 34.3% and 30.0%, respectively, of the fastest 5 and 10 finishes of all time. This is a dominant showing, exemplified by the fact that the population of Kenya (∼57.500 million) constitutes only ∼0.7% of the world population (∼8.232 billion) (https://www.unfpa.org/data/world‐population‐dashboard). What physiological, biomechanical and/or anthropometric marvels underlie this remarkable phenomenon?

Joyner (1991) predicted a potential marathon time that was ∼2 min less than 2 h and ∼9 min faster than the world record at that time. His modelling included a performance oxygen uptake (${\dot{V}}_{O_{2}}$), determined jointly by maximal oxygen uptake (${\dot{V}}_{O_{2}\max}$) and lactate threshold (LT). Jones (2024) has recently proposed refinements to Joyner's modelling. His edits include a consideration of critical speed, for which the maximal lactate steady state is an approximate physiological equivalent, and the new concept of resilience. Critical speed delineates the speed below which physiological variables can be stabilized and exercise can be sustained. In contrast, speeds faster than the critical speed do not permit stabilization, and each variable deteriorates to the point of exhaustion (Poole et al. 2021). For endurance exercise, Jones (2024) defined resilience as ‘the ability to resist fatigue and maintain performance’. Such resistance and maintenance would be exemplified by an athlete experiencing very little decline in their critical speed over the course of their event. Importantly, both Joyner (1991) and Jones (2024), in addition to many others (e.g., Conley & Krahenbuhl, 1980), have included running economy as a key fourth component of distance running performance.

In the search for an explanation of the endurance running success of Kenyans, ${\dot{V}}_{O_{2}\max}$ and maintenance of a high fractional utilization of the ${\dot{V}}_{O_{2}\max}$ do not appear to be distinguishing elements. It is in this context that Larsen et al. (2026) present key data in this issue of *Experimental Physiology* supporting a plausible explanation (slender lower legs) for the distance running success of male Kenyans. In elite Kenyan and Danish runners, Larsen et al. (2026) assessed ${\dot{V}}_{O_{2}\max}$, steady‐state ${\dot{V}}_{O_{2}}$ at two or three running speeds and various anthropometric measures of the lower leg. Their primary finding was that the elite Kenyans had a better running economy than their Danish counterparts, expressed appropriately in allometric units as millilitres per kilogram^−0.75^ per kilometre, and most importantly, that this advantage was explainable by the more slender lower legs of the Kenyans, as indicated by lower‐leg cross‐sectional area. When the running economy was adjusted for lower‐leg cross‐sectional area and relative lower‐leg length (lower‐leg length divided by total leg length), there was no difference between Kenyans and Danes. Importantly, when the testing conditions are considered, it is our opinion that there was no difference in ${\dot{V}}_{O_{2}\max}$ between the two groups.

Beyond the most basic unique finding discussed above, there are several other notable qualities of this manuscript.

- The elite athletes in this study were indeed elite. Both the Kenyan and Danish groups were ranked amongst the top 10 in their home countries at the time of the study. The Kenyans included three Olympic champions and one World champion. The Danes included five national record holders and a London Marathon winner.
- The finding of smaller lower legs in the Kenyans and their relationship to running economy amongst elite runners was bolstered by similar data in Kenyan and Danish sub‐elite runners (competitors who trained for ≥80 km per week and were able to run 10 km in <35 min).
- Even greater credence accrues because the same results were apparent in untrained Kenyan and Danish adolescents.
- In the sub‐elite and adolescent groups, the smaller lower‐leg size in Kenyans was confirmed not only by cross‐sectional area but was verified by volume measurements using water displacement.
- The authors were attentive to the accuracy of their data. Two specific measures are exemplary: (1) treadmill speed was verified directly with a measuring tape and timing of 10 revolutions whilst the participant was running on the treadmill; and (2) a steady state of ${\dot{V}}_{O_{2}}$ was verified during the submaximal runs for determination of economy.

In summary, Larsen et al. (2026) provide data deserving careful consideration in the discussion of underlying causes for Kenyan male dominance in distance races. Their results also accentuate the need for a similar study of Kenyan females. Similar to Kenyan males, Kenyan females have accomplished 34.3% of the 5 fastest finishes and 25.7% of the 10 fastest finishes in these same races (www.worldathletics.org). Do they also have a superior running economy underpinned by a smaller lower‐leg size?

## AUTHOR CONTRIBUTIONS

L. Bruce Gladden and Alexander C. Berry conceived the concepts for this Viewpoint. L. Bruce Gladden wrote the first draft. Both L. Bruce Gladden and Alexander C. Berry edited the draft and approved the final submission. Both authors are responsible for the intellectual content.

## CONFLICT OF INTEREST

None declared.

## FUNDING INFORMATION

None.

## References

[eph70166-bib-0001] Conley, D. L. , & Krahenbuhl, G. S. (1980). Running economy and distance running performance of highly trained athletes. Medicine & Science in Sports & Exercise, 12(5), 357–360. 7453514

[eph70166-bib-0002] Jones, A. M. (2024). The fourth dimension: Physiological resilience as an independent determinant of endurance exercise performance. The Journal of Physiology, 602(17), 4113–4128. 37606604 10.1113/JP284205

[eph70166-bib-0003] Joyner, M. J. (1991). Modeling: Optimal marathon performance on the basis of physiological factors. Journal of Applied Physiology, 70(2), 683–7. 2022559 10.1152/jappl.1991.70.2.683

[eph70166-bib-0004] Larson, H. B. , Boit, M. K. , Adamsen, M. L. , Madsen, F. , Berg, R. M. G. , & Hanel, B. (2026). Running economy and lower limb anthropometry in adult male Kenyan and Danish middle‐ and long‐distance runners and untrained adolescents. Experimental Physiology, 111(4), 1919–1932.41299215 10.1113/EP092758

[eph70166-bib-0005] Poole, D. C. , Rossiter, H. B. , Brooks, G. A. , & Gladden, L. B. (2021). The anaerobic threshold: 50^+^ years of controversy. The Journal of Physiology, 599(3), 737–767. 33112439 10.1113/JP279963

